# Ulcère de Lipschütz: une cause rare et sous diagnostiquée d'ulcération génitale

**DOI:** 10.11604/pamj.2013.15.43.2506

**Published:** 2013-06-07

**Authors:** Wafae Raffas, Badreddine Hassam

**Affiliations:** 1 Service de Dermatologie, CHU Ibn Sina, Université Med V, Souissi, Rabat, Maroc

**Keywords:** Ulcère, ulcère de Lipschütz, Epstein-Barr virus, adolescente, ulcer, Lipschütz ulcer, Epstein-Barr virus, teenager

## Image en médicine

L'ulcère aigu de la vulve ou ulcère de Lipschütz porte le nom de l'auteur qui l'a décrit initialement en 1913. Cette entité rare et probablement sous-diagnostiquée survient chez l'adolescente ou la jeune fille le plus souvent vierge au décours d'une affection fébrile. L'étiologie est inconnue, elle serait la conséquence d'une réaction immunologique exagérée à un agent infectieux tel que l'Epstein-Barr virus. Il s'agit d'une ulcération vulvaire aphtoïde unique ou multiple, hyperalgique, creusante et nécrotique d'évolution aigue, non sexuellement transmissible. La guérison spontanée survient en deux semaines parfois au prix de mutilations. C'est un diagnostic d'exclusion à retenir après avoir écarté les infections sexuellement transmissibles (IST), les causes auto-immunes, les traumatismes et autres étiologies des ulcérations génitales aigues. La prise en charge thérapeutique est symptomatique à base d'antalgiques, d'anesthésiques et de corticoïdes locaux, ou d'antibiotiques. Les cas sévères avec douleur et dysurie importantes peuvent nécessiter une hospitalisation et un sondage urinaire. Nous rapportons le cas d'une adolescente de 16ans, sans antécédents notables, sans notion de rapports sexuels. Elle consultait pour un syndrome pseudogrippal accompagné d'ulcères vulvaires creusants douloureux disposés en miroir. Il n'y avait pas de lésions de la muqueuse buccale. Des adénopathies sensibles étaient retrouvées à la palpation des aires inguinales. Les sérologies des IST étaient négatives, de même que les sérologies HSV, EBV et CMV. Une biopsie cutanée objectivait une ulcération avec des remaniements inflammatoires non spécifiques riches en polynucléaires neutrophiles. L'évolution sous antalgiques et soins locaux était favorable en dix jours sans séquelles ni récidives.

**Figure 1:**
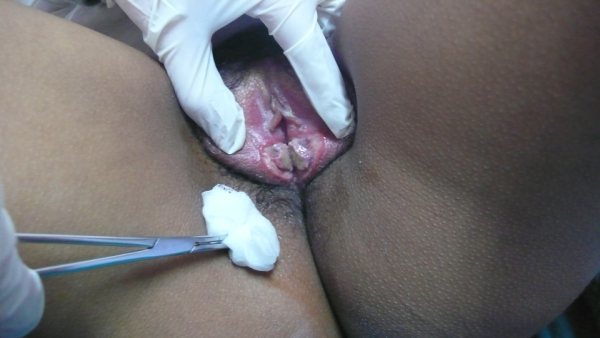
Ulcérations vulvaires en miroir (kissing ulcers). Deux ulcérations à fond propre de 25 mm de diamètre au niveau des petites lèvres, à disposition symétrique et en miroir par rapport à la ligne médiane

